# Ketone body supplement label claims: what supplement has been supplemented?

**DOI:** 10.17159/2078-516X/2019/v31i1a6369

**Published:** 2019-01-01

**Authors:** D Da Costa, V Bangalee, K Subban, R Naidoo

**Affiliations:** 1 Discipline of Biokinetics, Exercise and Leisure Sciences, University of KwaZulu-Natal, College of Health Sciences, South Africa; 2 Prime Human Performance Institute, 44 Isaiah Ntshangase Road, Durban, South Africa

**Keywords:** athletic performance, nutritional ketosis, *β*-hydroxybutyrate (*β*HB)

## Abstract

**Background:**

There is a keen interest in performance-enhancing supplementation and the associated benefits, despite reports of incorrect label claims made by manufacturers and the questionable efficacy of the supplements. The use of ketone body supplements as a source of fuel during exercise and sporting performance, in particular, is of interest to sportspeople. By increasing blood ketone body levels, with an accompanying decrease in blood glucose, may indicate a state of nutritional ketosis, whereby the body no longer relies on glucose metabolism but rather the metabolism of ketone bodies. This could be beneficial for long, slow steady-state endurance exercise.

**Discussion:**

There are numerous ketone body supplements on the market manufactured in South Africa and internationally. However, unlike medicines, the sports supplementation industry is poorly regulated. Furthermore, ketone body supplementation with regard to its effects on improving exercise and athletic performance is still unconvincing.

**Conclusion:**

Within the ever-changing sports supplementation industry, ketone body supplements are being used despite controversies regarding the accuracy and scientific merit of label claims. The ingredients and their quantities, as well as the performance benefits, need to be objectively validated.

Participants in recreational and competitive sports are constantly seeking ways to improve their performance. The widespread use of dietary supplements and their associated benefits in sporting performance has always been a topic of interest. Many of these products contain ingredients in varied mixtures and quantities. It is well documented that nutrient manipulation^[1–3]^ can increase aerobic exercise performance. Traditionally, supplementation focussed on glucose as a fuel source for increasing performance. More recently, awareness has shifted to the role of ketone body supplements to increase performance.

Ketone bodies can be endogenous where they are produced by the body in the liver, or exogenous, where they are taken in the form of a supplement ^[4]^. Ketone body supplements are commonly composed of β-hydroxybutyrate (*β*HB) combined with a salt, such as potassium, calcium or sodium (Na-*β*HB)^[4]^, which are then known as ketone salts. A ketone ester, otherwise known as a ketone monoester, such as (R)-3-hydroxybutyl (R)-3-hydroxybutyrate, is salt-free^[5]^ and not commercially available.

Ketone bodies, namely acetoacetate (AcAc), acetone, and *β*HB are lipid-based organic compounds produced by the liver during times of starvation and nutritional manipulation^[3]^, such as a high-fat, low-carb (HFLC) diet, also known as a ketogenic diet^[6]^. Increasing blood ketone body levels, with accompanying decreases in blood glucose may indicate a state of nutritional ketosis, whereby the body no longer relies on glucose metabolism but rather the metabolism of ketone bodies^[2]^. Following a ketogenic diet is not always easy, hence convenient alternative methods were introduced, such as the consumption of drinks containing exogenous ketone supplements.

Modern ketone supplement manufacturers claim to cut out this nutritional manipulation period with the ingestion of a supplement containing on average 12g *β*HB^[4]^. This amount can be supported by studies^[1–3]^ showing that by ingesting a ketone supplement, can result in ketosis in 30 minutes. Theoretically, increasing ketone body production via the decreased availability of glucose in the body, the ketone bodies become the alternative fuel source necessary for the brain and muscles of the body^[4]^. Thus, it is suggested that ketone supplementation has potential positive effects on exercise metabolism and performance in recreational and competitive sportspeople. However, these claims are based on limited studies^[1]^.

A study conducted by Clarke et al.^[7]^ found that the ingestion of ketone esters taken in a fasted state showed modest power output improvements in 30 minutes of rowing (averaging ~1% and up to ~2%) in 22 sub-elite male and female athletes. A similar study conducted by Cox et al.^[8]^ on 39 endurance athletes showed that the ingestion of ketone esters resulted in decreased lactate production with an average lactate concentration value of ~2–3 mmol (~50%) lower compared to a carbohydrate supplement. This decrease in lactate production could result in an increase in the performance of cyclists^[8]^. Although both studies showed positive reactions to the esters, they are very expensive and not commercially available. Furthermore, both studies had small sample sizes.

It has been suggested that ketone body metabolism may reduce the use of blood glucose and muscle glycogen, which could be beneficial for long, slow steady-state endurance exercise. Although glycogen sparing may be beneficial for endurance in long duration events, such as cycling, there are periods of high intensity where glucose is needed as the primary substrate. This suggests that ketone body supplementation may in fact lower the capacity to sustain high intensity efforts during endurance races. Two studies conducted on cyclists have shown that both ketone salts^[9]^ and ketone esters^[10]^ result in a decrease in power output and cadence. Furthermore, Rodger^[3]^ showed that the ingestion of a ketone body supplement by 12 highly-trained cyclists increased the respiratory exchange ratio (RER) and VO_2_ values at sub-maximal levels but failed to increase power output in four minutes of cycling^[3]^. In addition, Short^[11]^ found an increase in cycling performance in 12 recreational athletes following the ingestion of a ketone body supplement containing both *β*HB and caffeine (caffeine being a known performance enhancer). Thus it could be argued whether it was either the caffeine or the *β*HB that induced an effect on performance.

## Discussion

It is evident that the label claims of ketone body supplements on their effects with regard to exercise and sports performance are unconvincing and unsuccessful when tested in real-life situations^[3, 11, 9]^. Studies on ketone body metabolism during exercise are limited and there is no conclusive evidence supporting this for exercise and athletic performance enhancement^[6]^. Furthermore, particular attention needs to be paid to the ingredients in these supplements and their respective quantities. Unlike medication, supplementation is poorly regulated internationally, as premarket approval by bodies such as the Food and Drug Administration (FDA) and the South African Health Products Regulatory Authority (SAHRPA), with a few exceptions, unnecessary. Thus the regulation of performance-enhancing supplementation is often entrusted to their distributors and manufacturers to ensure that these products are both safe and effective. SAHPRA monitors certain but not all performance-enhancing supplements in South Africa, leaving the majority of both local and international supplements unregulated^[12]^.

Studies conducted on ketone body metabolism have repeatedly stated the adverse effects of ingesting ketone body salts and or esters^[4, 6, 10]^. Some of these effects include gastrointestinal tract discomfort, nausea, diarrhoea and abdominal pain, which may counteract any additional benefits associated with higher doses of these products^[3]^. A study by Veech^[5]^ found that only ketone esters should be used, as increased ketone salts could result in acidosis. Another study^[4]^ found that if following the recommended daily serving sizes, the recommended daily allowances of minerals found in most supplements were up to five times higher than necessary, potentially causing metabolic alkalosis, a condition in which the pH of tissue is elevated beyond the normal range^[4]^.

Apart from the documented adverse effects, attention also needs to be given to the costs of ketone body supplements. Pure *β*HB salts cost approximately $235 (R3370) for 50g. This converts to approximately $67 (R960) per 12g of *β*HB salts, yet wholesale ketone body supplements containing up to 12g of *β*HB salts per sachet are being sold for as little as $2.80 (R40) ^[4]^. The mixture of ingredients and related quantities of each ingredient needs to be queried.

## Conclusion

As recreational and competitive sportspersons are continually seeking new ways to increase their performance, it is apparent that dietary supplementation is a popular method of choice^[1]^. Despite the negative side effects and the limited evidence-based research on exercise and athletic performance, together with inaccurate supplement label claims, ketone body supplements continue to be used among sportspeople.

The accuracy and scientific merit of the product label claims are controversial. The ingredients and their quantities, as well as their performance benefits, need to be objectively validated. Furthermore, if the composition of the supplement is questionable, then the validity of the research on selected dietary supplements is compromised.
